# Prenatal maternal docosahexaenoic acid intake and infant information processing at 4.5mo and 9mo: A longitudinal study

**DOI:** 10.1371/journal.pone.0210984

**Published:** 2019-02-13

**Authors:** Alison Rees, Sylvain Sirois, Alison Wearden

**Affiliations:** 1 Department of Psychology, Lancaster University, Lancaster, United Kingdom; 2 Département de Psychologie, Université du Québec à Trois-Rivières, Trois-Rivières, Canada; 3 School of Psychological Sciences, The University of Manchester, Manchester, United Kingdom; University of Illinois, UNITED STATES

## Abstract

Previous research suggesting an association between maternal prenatal docosahexaenoic acid (DHA) intake and infant cognition has yet to assess whether there is a critical trimester for the observed effects. We used a comprehensive Food Frequency Questionnaire to estimate DHA levels during both the second and third trimesters of pregnancy, in a sample of 125 pregnant women. Infants were assessed at 4.5 months and 9 months post-partum using specific tests of visual acuity, habituation, and visual attention. Based on maternal DHA levels during pregnancy, mothers were subdivided into high, medium, and low groups, and their infants compared for task performance using one-way ANOVAs with maternal DHA groups. On the 9 month visual acuity test, infants whose mothers were in the medium DHA group performed significantly better than those with mothers in the low or high DHA groups (p = 0.008). However, no significant finding was found for any of the other cognitive assessment measures. Despite a number of studies reporting a positive effect of higher DHA levels on cognitive development, this study fails to support those conclusions. We can, however, conclude that it appears to be DHA intake in the third trimester specifically, which is influencing the development of visual acuity towards the end of the first postnatal year.

## Introduction

Long-chain Polyunsaturated Fatty Acids (LCPUFAs), especially those of the Omega-3 (n-3) family, play a vital role in neurological functioning, being highly concentrated in the myelin sheath and membranes of synaptic terminals of the brain and central nervous system, (CNS)[1,2] and play an important role in the development and control of neurotransmitter systems[3,4]. Omega-3 LCPUFAs are believed to be particularly important during the prenatal and early postnatal developmental periods[5,6,7] with docosahexaenoic acid (DHA, n-3 family) appearing to be the key nutrient at this time. DHA is preferentially transferred across the placenta and selectively accreted in fetal brain and retinal tissue during pregnancy[8,9], with rapid accretion and uptake beginning at the start of the third trimester, continuing throughout this trimester, and into the first two years of postnatal life, albeit at a slightly reduced rate postpartum. This characteristic pattern of preferential placental transfer, accretion and uptake suggests a particular need for this nutrient within the developing brain at that time[10].

The fetus acquires its nutrients from the mother; therefore, the maternal diet is key in determining the levels of DHA available to the fetus. DHA (along with a number of other n-3 LCPUFAs) can be synthesised from the n-3 precursor Essential Fatty Acid (EFA) alpha linolenic acid (ALA). The term ‘Essential’ refers to the inability of the body to synthesis this nutrient *de novo*, hence ALA must be obtained directly from the diet. Once present in the body however, the conversion process of ALA to longer chain PUFAs within the n-3 family, such as DHA (occurring via enzymatic processes of desaturation and elongation) is poor in humans,[11] although slightly more efficient in females [12] and possibly upregulated in women of child-bearing age and during pregnancy[13]. Individual metabolic factors and genetic variations (specifically within the FADs gene clusters) also affect metabolism of LCPUFAs, including DHA[14,15]. Fortunately, DHA is available in pre-formed state directly from the diet, although food sources containing meaningful amounts are limited. DHA is found in its highest concentrations in oily fish such as sardines, salmon, and mackerel, with a number dietary supplements containing DHA (for example, fish oil or algal supplements) also available.

Prenatally, therefore, the developing fetus, with its need for DHA, is reliant on maternal dietary intake of this nutrient during pregnancy via food ingestion or supplementation. Postnatally, breastmilk contains DHA (in varying amounts, largely dependent on the maternal diet [16]) and, in breastfed infants, provides a pre-formed source of DHA to the infant brain. As the development of cognitive functions is so rapid during infancy and early childhood, it seems plausible to suggest that varying levels of these nutrients may have an effect on cognitive development at these stages.

The majority of early studies examining DHA levels and cognitive development focused on postnatal DHA intake, most often through the manipulation of DHA levels in infant formula, but also through alterations in breastmilk DHA. More recently however, as the importance of the prenatal environment has become increasingly recognised,[17,18] attention has turned to focus on prenatal maternal intake. Alongside this, a recognition of the importance of selecting the most appropriate cognitive tests when assessing nutritional impact on cognitive development, has led to a shift away from the use of global assessment measures to examination of specific cognitive systems[19,20,21]. Use of, for example, the Bayley Scales of Infant Development (BSID), is now largely accepted as being inappropriate for these sorts of studies. Rather, when examining the potential effects of specific nutrients, which likely act within specific cognitive domains, examination of individual cognitive systems is needed.

### Rationale for, and development of, the current study

This study assessed infant cognitive development in the areas of visual acuity, habituation, and visual attention (using a novel attention task designed to measure not just sustained attention but also attentional shift and release ability) in relation to prenatal DHA intake. Due to the high concentration of DHA in the retina, visual acuity is perhaps the most studied outcome measure in relation to DHA levels, but there is still an inconsistent pattern of findings. The majority of studies report improvements in visual acuity (at least at some ages) with increased levels of DHA[22–24] but others reporting no benefit[25–27] with no comparisons currently having been made between second and third trimester intake.

Habituation is utilised frequently in infancy research, and habituation rate and looking time patterns across trials have been suggested as being reflective of infants’ information processing abilities, with faster habituation (and shorter mean looking time across the early trials in a task) representing faster information processing. A number of studies have looked at habituation in relation to DHA levels with some reporting positive associations between higher DHA levels and infant habituation[28,29] and others reporting no effect[30,31].

There are a number of indications suggesting that attention may be one of the key cognitive systems affected by differing DHA levels. This comes not just from research examining DHA levels in relation to normal infant performance in attention tasks, but also from preliminary, suggestive findings that low DHA levels (or low LCPUFA metabolism) may play a part in developmental disorders such as attention deficit disorder (ADD), of which impairments in the attentional system are a feature. A number of studies have shown better infant and child performance on tasks measuring attention in relation to higher DHA levels [28,32,33].

The study employed a comprehensive Food Frequency Questionnaire (FFQ) to provide estimated intake levels of DHA for both the second and third trimesters of pregnancy. Owing to the differential development that takes place in the second and third trimesters of pregnancy, this study uniquely aimed to assess whether there is a critical time point during which LCPUFA levels, specifically DHA, affect infant cognitive development. By using the FFQ method of dietary data collection the study also had the advantage that any association which may be shown between dietary DHA intake (for example with increased consumption of oily fish) and improved infant cognitive development will be more able to inform public health recommendations for pregnant women. As individual fatty acid metabolism will vary substantially, showing a direct link to improvements in cognitive performance based on maternal dietary intake should allow dietary recommendations to be made which are more universal than those based on research linked solely to individual physiological fatty acid profiles (e.g., blood-plasma studies).

Infants were tested at two time points during the first year of life, these being 4.5 month and 9 months, which were selected as being developmentally appropriate ages for the assessments and for maximum infant cooperation during the testing sessions.

### Hypotheses

Our hypotheses were:

- Higher maternal DHA intake during pregnancy will result in better infant performance on tasks of visual acuity, habituation and visual attention at 4.5 and 9 months post-partum. In the case of habituation, faster habituation will be taken as indicative of better performance. For visual attention, two measures will represent better performance: greater sustained attention ability, and a quicker response time to switch attention to a secondary competing stimulus when it appears.
- If there is a critical prenatal time point at which DHA intake results in significantly better performance in the above tasks, this will be related to third trimester intake when DHA accretion and uptake in fetal brain is rapid[10].

## Materials and methods

### Participants

A total of 125 expectant mothers were recruited for the study from a catchment area within an approximate 30 mile radius from the University of Manchester. Both primiparous and multiparous women took part in the study. Recruitment strategies included leafleting at numerous venues; media coverage; features and adverts in local magazines and newspapers; recruitment emails aimed at University staff and students; and a website dedicated to the study. Women were recruited on the basis that they were experiencing a single (i.e., no twins, triplets, etc.), healthy pregnancy, were at least 12 weeks pregnant, and had no significant health problems. Health problems leading to exclusion from the study were: health problems requiring any degree of longer-term treatment, any chronic conditions, anything that could potentially impact on the pregnancy, or anything requiring long-term medication. The first data collection point was after the 20 week scan (at which any major fetal abnormalities would be detected) had taken place.

This study was conducted according to the guidelines laid down in the Declaration of Helsinki and all procedures involving human participants were approved by the University of Manchester’s School of Psychological Sciences Research Ethics Committee (code 30/07P). Written informed consent was obtained from all participants at each stage of the study.

### Design

**Fig 1:**
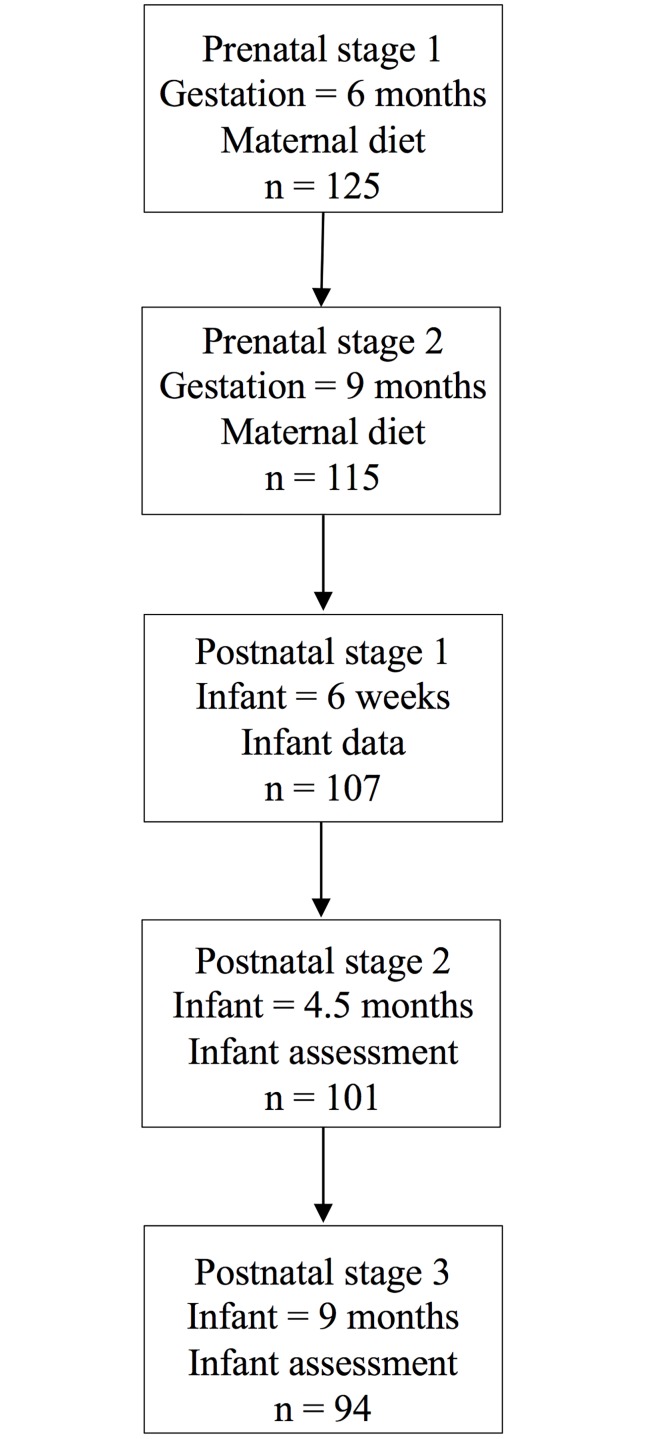
Illustration of the data collection points of the study.

A longitudinal study design was employed with data collection points as shown in Fig 1. Prenatal stages focused on maternal dietary data collection and postnatal stages on infant data collection and cognitive assessment.

### Procedure, materials and apparatus

#### Prenatal stage one: Gestation = 6 months

Participants completed the first stage of the study between their 21st and 28th week of pregnancy. At this visit 93 (74.4%) mothers were assessed at home and 32 (25.6%) at the University of Manchester Babylab. Mean gestation at prenatal stage 1 was 24.56 weeks (range = 21–28 weeks, SD = 2.10); total n = 125.

***Maternal questionnaire measures***. Maternal dietary information was obtained using a Food Frequency questionnaire (FFQ), the Diet History Questionnaire (DHQ, developed by the National Institute for Cancer, NIC: US) which has been shown in a number of validation studies [34] to provide reasonable nutrient estimates for a range of n-3 and n-6 PUFAs. The DHQ has 149 questions that focus on frequency of consumption and approximate portion sizes of various foods and drinks. Detailed information was given to enable mothers to accurately estimate portion size. The DHQ was adapted for use with a UK sample group and to enable use for a three month (i.e., a single trimester) period. The DHQ also contains a number of questions relating to the use of dietary supplements, such as vitamins and minerals, fibre, herbal and other supplements, including fish oil supplementation.

Alongside the DHQ, a number of additional dietary questions were asked, specifically in relation to fish intake. Five supplementary questions focused on fish intake over the past three months, and also asked about oily fish separately to other types of fish. These were taken from a Project Viva FFQ which has been validated (via erythrocyte concentrations) as providing accurate estimates of n-3 LCPUFAs in early pregnancy.[35] These additional questions have been used in an earlier study that assessed fish intake in pregnancy in relation to infant cognitive development. [36]

#### Prenatal stage two: Gestation = 9 months

The initial procedure at this visit was identical to that of the 21–28 week gestation. Ninety-nine (79.2%) mothers were assessed at home and 16 (20.8%) at the Babylab (total n = 115). Mean gestation at Prenatal stage 2 was 36.15 weeks (range = 35–37 weeks, SD = 0.84).

***Maternal questionnaire measures***. Data collection at this stage was similar to the previous stage. Participants were reminded that dietary questions related to their consumption since the previous visit.

An additional questionnaire was administered asking about any PUFA supplements that may have been taken, such as fish oils, algal oils or omega blends, both before and during pregnancy. Data on frequency, duration and manner of supplementation were obtained, as well as product information, in order to enable the supplemented nutrient levels to be calculated. Where possible the supplement container was examined and the relevant nutritional information recorded.

At the end of the visit participants were given two forms in a self-addressed envelope to be returned to the researcher following the birth of their baby. This included a consent form for mothers to indicate that they were happy to progress to the post-partum stages of the study and a questionnaire about their newborn. If these forms had not been received three months past each participants’ expected due date then a reminder letter was issued and, if this failed to generate forms being returned or a request for lost forms, it was assumed that the participants no longer wanted to continue on with the study and no further contact occurred.

#### Postnatal stage one: Infant = 6 weeks

Within 6 weeks post-partum mothers returned the questionnaire which provided initial data about their newborn. This was the earliest point of data collection relating to the infant. The questionnaire asked about infant birth-weight, head circumference, length, sex, birth date, health, and any problems that may have occurred during labor. Data was received for 107 infants at this stage of the study.

#### Postnatal stage two: Infant = 4.5 months

All infant assessment took place at the Babylab at the University of Manchester. When booking appointments, mothers were asked to bring their infants during the times when they felt they would be most alert and content (i.e., not hungry or tired), and if babies were unwell on the day of testing mothers were encouraged to re-schedule the appointment. The mean infant age at this postnatal stage was 142.0 days (4.6 months) with a range of ages from 117–153 days (3.8–4.9 months; SD = 8.92 days); total n = 101.

***Infant cognitive assessment***. At postnatal stage 1, all infant-derived cognitive data was gathered using the same general procedure. Infants were placed in a high chair and securely strapped in. Once the infant appeared comfortable and content, the high chair (which was fixed onto castors to allow the infant and chair to be moved around with ease) was wheeled into the testing booth. The booth consisted of two large felt boards positioned in parallel with the corner walls of the lab which resulted in a cubicle of roughly 6 feet by 3 feet, with an opening into which the high chair could be wheeled. Inside the cubicle was a Dell computer monitor (36cm x 28cm screen size) on which task stimuli were displayed, a Tobii x50 eye tracker positioned under the monitor, and a video camera which was used to provide live feed to a monitor outside the cubicle which allowed mothers and the experimenter to view infants as they carried out the tasks. As well as strip lighting in the lab, extra lighting was provided in the testing cubicle by two light bulbs positioned on a rail at the top of the cubicle.

As infants were wheeled into the cubicle an attention-grabbing video of a popular children’s animation was played on the monitor along with a cheery, catchy musical tune. This focused the infants’ attention onto the monitor whilst positioning them in the correct place for the eye tracker and prevented infants growing bored and restless whilst this was taking place. At the side of the monitor scene, a small eye gaze capture window was visible which was used to guide the positioning of the infant to the correct position for the eye tracker. Correct positioning was achieved when both eyes were visible at the centre of the eye gaze capture window. At this point the attention-grabbing animation was stopped and the infants’ eye gaze was calibrated (using a 5 –point calibration sequence) to the eye tracker. Once calibration was completed, the three tasks were carried out in the order given below. During all tasks mothers were instructed not to interact with or encourage their infants and the testing occurred in silence.

***Visual acuity***. This was assessed using paired presentation of visual acuity gratings and uniform grey squares. The side to which the grating or the grey square appeared was random over trials to control for any preferential looking based on position by the infant. The acuity gratings began with four stripes creating a grating within one of the squares and gratings reduced in size to a potential minimum of 1024 stripes creating a grating within the square. The grating square and uniform grey square were designed with the property of constant luminance so overall luminance in each square was equal. Gratings were presented in a in a two-step up, one-step down pattern. If infants did not look at the screen for 2 concurrent presentations the task automatically stopped.

***Habituation***. A visual preference paradigm was used in order to assess habituation rate. This involved repeated paired presentations of a familiar and novel stimulus composed of different patterns of coloured dots. The familiar stimulus, presented each time, consisted of 4 yellow dots arranged in the shape of a square. Novel stimuli included patterns of 2, 3, 4 or 5 dots in either blue or red. The side of presentation of the novel or familiar stimulus was random. If infants did not look at the screen for 2 consecutive trials the task was automatically stopped. Otherwise, the habituation criterion was taken to be half of the average looking time to the familiar stimulus on the first three trials. This was assessed at the end of each trial (beginning at trial six) from the average of the previous three trials. The number of trials the infant required to do this was taken as their individual habitation rate.

***Visual attention***. This task consisted of 4 consecutive presentations of an engaging stimulus to the infant. The stimulus was a circular, yellow, smiley face (approximately 6cm diameter) which rotated around the monitor on a circular path (of approximately 30cm diameter). The eye tracker recorded the tracking of this object by the infant as it rotated on its path. On each trial there was a randomly selected critical position on the circular path (at 0°, 90°, 180°, or 270°) and if the infant was able to continually track the stimulus for the 10 degrees preceding the critical position then a second stimulus would appear in the centre of the screen. This stimulus was a 3D blue box (approximately 5cm length and height). The original smiley face stimulus continued to move on its path despite the appearance of the second stimulus. Measurements were taken as to whether the infant shifted their attention to the new stimulus and, if they did, how long it took them to do this. If infants remained fixated on the original stimulus both stimuli remained on the screen for a further 4 rotations of the original stimulus and then both disappeared and a new trial began. There were four trials in total and if the infant did not make the box appear during any one trial after 9 rotations of the face the next trial began.

#### Postnatal stage three: Infant = 9 months

The procedure followed was identical to that outlined above. The mean age at postnatal stage 2 was 295.7 days (9.54months). The range of ages was 276–327 days (8.9–10.5 months; SD = 12.3 days); total n = 94.

***Infant cognitive measures***. The visual acuity, habituation and attention computer based tasks from the 4.5 month visit were again completed by infants at this stage of the study in line with the procedures detailed above.

***Maternal questionnaire measures***. The quality of the home environment is potentially a significant confounding factor impacting on infant cognitive development. The Home Screening Questionnaire (HSQ)[37] was used to provide a measure of home environment at 4.5 and 9 month assessments. It included questions relating to the amount of stimulation the infant receives in the home, any discipline practices, how often the infant is taken out, etc. Mothers completed the HSQ at both infant testing sessions. The HSQ has been successfully used in a number of similar studies[38] and is provided with detailed scoring criteria providing an overall score from 0–43.

Mothers also completed a breastfeeding questionnaire at both visits which included a question on the duration for which their infant had been breastfed. As breastfeeding has been shown in some studies[39–41] to positively affect infant cognitive development this data was collected to assess breastfeeding duration as a potentially confounding factor in the analyses.

Any mothers who had breastfed their baby for 5 days or longer also completed the DHQ at the 4.5 month infant visit. Those who continued to breastfeed after the 4.5 month visit again completed the DHQ at the 9 month visit. As there have been reports of postnatal LCPUFA levels affecting cognitive development it was important to have a measure of the maternal postnatal intake in those who were breastfeeding (and thus providing LCPUFAs via breastmilk to their infants). If infants received any formula the mothers filled out a questionnaire providing details of the formula brand, frequency and amount of consumption.

### Data processing

As a number of the participants in the study were taking fish oil supplements containing meaningful amounts of DHA the values used for these nutrients in all analyses consisted of the *total* DHA amounts; i.e., the sum of the supplemental intake (calculated per day, for each individual participant, using values from the specific brand used and the number of times taken per day or per week) and the DHQ estimate for dietary daily values.

The dietary data files collected from the DHQ were converted to nutrient files following the prescribed procedure using the Diet*Calc software package provided by the NIC for this purpose. This procedure generated a file which contains daily nutrient (including DHA) and food group estimates for each participant at each point of testing.

### Analysis plan

Initial inspection of the data took place and any highly non-normal data were transformed, in this case, the visual acuity data. The data were examined for potential covariates and covariate analyses and post-hoc analyses performed. All analyses were run with DHA (total) as the independent variable, and a number of cognitive outcome measures, in accordance with the study hypotheses set out in the introduction. Separate analyses examined the effects of levels of the nutrients in the second and third trimesters. Participants’ individual nutrient values were recoded into 3 equal-sized groups: low, medium and high, by calculation of tertile groups. DHA values which fell within each group, for each trimester, were as follows: second trimester low (range = 0.000g–0.050g, M = 0.022g, SD = 0.015g); second trimester medium (range = 0.060g–0.160g, M = 0.097g, SD = 0.030g); second trimester high (range = 0.170g–0.460g, M = 0.300g, SD = 0.083g); third trimester low (range = 0.000g–0.040g, M = 0.018g, SD = 0.015g); third trimester medium (range = 0.050g–0.160g; M = 0.095g, SD = 0.031g); third trimester high (range = 0.180g–0.560g, M = 0.309, SD = 0.109g). In a number of preliminary analyses, one-way ANOVAs were used to check for homogeneity of the groups across a number of parental and infant characteristics. These are presented in Table 1. Comparisons were done for both second and third trimester data. Parental variables examined as potential covariates in the analyses were: maternal age; maternal and paternal years in education (taken as number of years in education since beginning secondary school; i.e., from age 11 years onwards); and pre-pregnancy Body Mass Index (BMI), which was calculated according to the formula: weight (kg) / height (m^2^). Infant variables examined were birth weight, length and head circumference; gestation length; and Apgar scores (recorded 1 minute and 5 minutes post-delivery). The mean duration of breastfeeding in each group was compared, along with the age of infants at testing, as a significant age difference between the two groups could feasibly impact on any findings. Finally, HSQ scores were also examined as a potentially confounding factor.

**Table 1:** Mean values of parental and infant characteristics for the high, medium, and low DHA groups used in the analyses. Comparisons of group means were carried out with one-way ANOVAs and the p values are given below.

	2^nd^ trimester				3^rd^ trimester			
	Low	Med	High	p	Low	Med	High	p
**Parent characteristics**								
Maternal age (yrs)	32.3	33.7	33.0	0.28	33.0	33.4	32.8	0.78
Pre-pregnancy BMI (kg/m^2^)	24.5	24.0	23.3	0.51	24.3	24.2	23.2	0.47
Maternal education (yrs)	9.75	10.6	11.0	0.12	9.78	10.8	10.8	0.12
Paternal education (yrs)	8.84	10.0	9.14	0.09	9.44	10.0	9.29	0.43
**Infant characteristics**								
Weight (kg)	3.73	3.55	3.66	0.23	3.73	3.51	3.73	0.05*
Head circumference (cm)	35.3	34.9	34.9	0.47	35.1	34.8	35.2	0.41
Length (cm)	53.3	53.6	53.3	0.90	54.6	52.9	53.7	0.16
Apgar score at 1 minute	8.64	8.68	8.33	0.62	8.77	8.62	8.25	0.41
Apgar score at 5 minutes	9.27	9.41	9.05	0.53	9.27	9.41	9.05	0.55
Age at 4.5m testing (days)	142	142	142	0.99	142	141	143	0.72
Age at 9m testing (days)	293	296	297	0.58	295	295	298	0.74
**Other factors**								
Breastfeeding duration (months)	10.4	9.38	10.8	0.78	11.3	8.68	11.4	0.31
HSQ score (4.5 months)	27.9	26.8	30.9	0.09	27.5	27.0	30.3	0.19
HSQ score (9 months)	35.0	34.2	36.5	0.02*	34.1	34.7	36.1	0.08

* p≤ 0.05

The data reported in this paper forms part of a larger longitudinal study, for which a number of additional measures were taken, including: maternal mood; general health; previous pregnancies; postnatal diet including solid food intake and fish consumption. There was also a further point of data collection, at 22 months postnatally, and it is anticipated that further follow up cognitive assessment will take place throughout childhood.

## Results

### Retention rate

Some participants dropped out of the study at each of its time points. Retention rates (relative to initial sample size) were as follows: second pregnancy visit = 92.0% (n = 115); 6 weeks post-partum 85.6% (n = 107); 4.5 month infant assessment = 80.8% (n = 101); 9 month infant assessment = 75.2% (n = 94). Reasons for leaving the study fell into the following categories: non-return of post-birth forms (n = 14); unable to be contacted despite repeated attempts (n = 6); moving away from the area (n = 5); premature birth (n = 2); changed mind about taking part (n = 1). Three participants were also lost due to the researcher being unavailable to test infants when they reached the appropriate age.

To check for any unintentional biasing as a result of dropout, the sub-sample of participants who dropped out was compared with the retained sample using one-way ANOVAs. It can be seen from Table 2 that at 4.5 months the groups differed with respect to maternal education (with the retained group having mothers with significantly longer years spent in education), paternal education (with the retained group having fathers with significantly longer years spent in education), and gestation length (with the retained group having a significantly longer gestational period than the dropout group). At the 9 month testing session, the two groups do not significantly differ on any parental or infant characteristics.

**Table 2:** Comparisons of variable measures between the dropout group and the retained group of participants at 4.5 and 9 months. The mean value for each group is given along with the p value for the comparison of group means.

	4.5 months			9 months		
	Dropout	Retained	p	Dropout	Retained	p
**Parent characteristics**						
Maternal age (yrs)	33.3	33.1	0.85	32.9	33.3	0.62
Paternal age (yrs)	35.4	33.9	0.27	34.6	35.3	0.55
Pre-pregnancy BMI (kg/m^2^)	25.1	23.7	0.12	24.2	23.9	0.66
Maternal education (yrs)	9.48	10.7	0.03*	9.90	10.7	0.13
Paternal education (yrs)	8.30	9.82	0.02*	8.73	9.80	0.06
**Infant characteristics**						
Gestation length (days)	275	283	0.03*	279	283	0.16
Weight (kg)	3.63	3.70	0.70	3.83	3.60	0.09
Head circumference (cm)	35.6	34.9	0.23	35.6	34.9	0.06
Length (cm)	53.0	53.6	0.77	53.8	53.5	0.83
Apgar score at 1 minute	8.40	8.59	0.75	8.33	8.61	0.55
Apgar score at 5 minutes	8.40	9.34	0.08	8.89	9.33	0.28

* p≤ 0.05

### Pregnancy and birth data

One hundred and seven questionnaires were returned at this stage of the study reporting 55 female infants born (51.4%) and 52 male (48.6%). Gestation length (in days) was calculated from the difference between the birth date and due date, with a standard pregnancy taken as lasting 280 days. The mean gestation length was 282.63 days (range = 238–296 days, SD = 8.85). Of 107 births, 86 (80.4%) were via normal vaginal delivery, 10 (9.3%) were delivered via elective caesarean, and 11 (10.3%) via emergency caesarean.

### Preliminary analyses

Table 1 shows that the high, medium, and low DHA third trimester groups used in the main analyses differed with regards to infant birth weight, with the medium group having a significantly lower birth weight compared to both the low and high groups. The second trimester groups differed with regards to their HSQ score at 9 months, with post-hoc tests revealing the medium group as having a significantly lower HSQ score than the high group. Both of these factors could potentially be associated with later cognitive development. Therefore, they will both be considered during the analyses, and included as covariates where appropriate.

### Main analyses

Table 3 presents the mean values for each of the infant cognitive measures, for the second and third trimester high, medium, and low DHA groups.

**Table 3:** Mean values for infant cognitive outcome measures for the high, medium, and low DHA groups used in the analyses. Comparisons of group means were performed with one-way ANOVAs and the p values are given below.

	2^nd^ trimester DHA				3^rd^ trimester DHA			
	Low	Med	High	P	Low	Med	High	p
**Visual acuity**								
4m highest grating	62.3	103.8	102.0	0.45	75.1	108.6	87.4	0.60
9m highest grating	139.8	205.7	119.4	0.16	119.0	230.4	98.6	0.008*
**Habituation**								
4m initial look duration	179.7	145.8	219.0	0.13	170.8	160.1	192.5	0.69
9m initial look duration	187.4	244.9	269.9	0.26	205.6	243.6	257.1	0.58
4m trial habituated	8.88	9.63	10.11	0.76	9.41	10.09	9.10	0.76
9m trial habituated	8.32	10.39	10.56	0.20	9.05	9.58	10.83	0.45
**Visual attention**								
4m no. box appearances	2.14	1.73	2.05	0.43	2.23	1.66	2.00	0.23
9m no. box appearances	2.25	2.07	2.13	0.94	2.21	2.00	2.07	0.90
4m rotations preceding box	2.45	2.19	2.21	0.59	2.34	2.25	2.27	0.95
9m rotations preceding box	2.73	2.23	1.99	0.26	2.85	2.14	1.98	0.12
4m switch response time	3745	3855	3341	0.95	3094	3841	4341	0.77
9m switch response time	2327	1209	1320	0.24	2050	1339	1341	0.54

* p≤ 0.05

### Visual acuity

The exponential acuity data were normalised to a linear scale using a log 10 transformation. Following this, a univariate ANOVA revealed a significant main effect of group (F [2, 72] = 5.02, p = 0.009, partial Eta squared = 0.122, observed power = 0.80; homogeneity of variances assumed). Post-hoc Bonferroni analyses revealed a significant difference between the low (1.64) and medium group means (2.12), p = 0.01.

As birth weight was seen in the preliminary analyses to be significantly different in the medium DHA group in relation to the low and high DHA groups further analyses were carried out to ensure that it was the DHA levels determining the differences observed in acuity performance rather than an effect of birth weight. Infant birth weight was found to be not significantly correlated with 9 month visual acuity (p = 0.06). When the ANOVA was repeated with birth weight entered as a covariate the effect of third trimester DHA was still significant in relation to 9 month visual acuity even after adjustment for birth weight (F [2, 71] = 3.95, p = 0.02, ηp2 = 0.10, observed power = 0.69).

#### Habituation

There were no significant differences on the habituation task between infants with mothers with low, medium and high DHA levels at either stage of pregnancy (see Table 3).

#### Visual attention

There were no significant differences on the visual attention task for infants with mothers with different levels of DHA at either stage of pregnancy (see Table 3).

#### Postnatal dietary data

While the focus of this study was to examine maternal prenatal nutrition in relation to infant cognitive development, it is also important to consider potential effects that the postnatal diet may be having. In order to check that any observed effects were not actually related to the postnatal, rather than the prenatal, DHA status of the infants, the postnatal dietary data obtained from the breastfeeding mothers (via the DHQ) and the formula questionnaires was also analysed following the procedures used for the prenatal data above. There were no significant findings for postnatal DHA intake in relation to any of the cognitive outcome measures.

## Discussion

This aim of this study was to examine the effect of maternal DHA intake during the second and third trimesters of pregnancy, on infant postnatal cognitive development. As a number of previous studies have reported improvements in performance on a number of cognitive tasks, for infants whose mothers had a higher DHA intake during pregnancy (or postnatally via breastmilk or DHA containing infant formula), our hypotheses predicted a similar pattern of results. However, for the two cognitive assessment measures we examined (habituation and sustained visual attention) we found no significant differences in infant performance at either 4.5 months or 9 months of age. Given that positive findings have been previously reported elsewhere[28,29,33] (although a number of null findings have also been reported [30, 31]), there could be a number of reasons for this. It may be that the levels of DHA among the women in our sample were not high/variable enough to elicit increased performance in the cognitive tests employed; that the cognitive tests themselves were not sensitive enough to pick up on any performance differences; that the age of infant testing sessions was not appropriate to detect what may, potentially, be transient effects on cognitive development; or, simply, that variations in prenatal maternal DHA intake do not have any significant effect on the development of infant attention or habituation performance. Or, more likely, that a combination of some/all of these factors are contributing to the observed results.

Alongside the habituation and attention tasks we also examined infant visual acuity. This task, although not strictly a measure of cognitive performance, is one of the earliest neural developmental systems which has been examined in relation to DHA, owing to the high levels of DHA in retinal tissue, a large amount of which is accumulated in foetal retinal tissue during the third trimester of pregnancy. Despite being well researched however, the same inconsistent pattern of findings as with cognitive outcome measures is apparent with visual acuity measures. Our results further add to this equivocal collection of data as we report an unusual pattern of performance for this task. We found that infants whose mothers were in the medium DHA intake group had the best performance in the visual acuity task at 9 months of age. Interestingly, and in support of our hypothesis relating to potential trimester specific effects, this finding was only significant in relation to third trimester DHA intake levels. Prenatal DHA accretion in the brain begins to rise rapidly at the start of the third trimester and it is preferentially taken up by the fetal brain[10,18] thus we had hypothesised that any trimester specific effects of maternal DHA intake would be related to third trimester intake, which appears to be the case.

The performance direction of the results themselves however, was not as expected. Infant task performance in the medium DHA group was significantly better than both the low DHA group *and* the high DHA group. On closer examination, it can be seen that the worst performance is the high DHA group, although the difference in performance between the low DHA group and the high DHA group is minimal. This contrasts with our hypothesis that higher DHA levels will result in better development of infant visual acuity, although even in previous studies reporting positive outcomes for visual acuity with increased DHA levels, it has not always been seen to be the case that the higher the levels, the greater the increase in performance. For example, a recent study, the DIAMOND (DHA Intake and Measurement of Neural Development) study[42] attempted to clarify if there is an optimum level of DHA in relation to visual acuity. Birch and colleagues[42] examined visual evoked potentials in four groups postnatally, given formula containing no DHA (control group); 0.32% DHA; 0.64% DHA and 0.96% DHA. All DHA supplemented formula also contained 0.64% AA. They found that each of the DHA groups had significantly better performance when compared with the control group; however, there were no significant differences between any of the DHA groups. This implies that there may be a threshold of sufficient DHA that is necessary for optimal performance on acuity tasks, but that there is not necessarily a linear relationship of greater DHA linked to greater performance. It appears that this group had sufficient DHA to perform optimally, and increasing the level further did not result in increased performance.

We feel that our reported pattern of results for visual acuity are currently unexplainable. It may be that this is a random, chance finding, despite the low likelihood of the finding under the null hypothesis (p = 0.008). Interestingly however, when looking at the 9 month old visual acuity data for second trimester DHA intake, while failing to achieve statistical significance (p = 0.16), the pattern of results is identical to those of the third trimester—with the greatest performance in the medium DHA group and worse performance for both the low and high groups (and again, the worst performance being seen in the high DHA group). The fact that the pattern of results is identical to that of the third trimester results gives more support to this not being a random chance finding. We feel that despite our being able to find no theoretical explanation for this pattern of results, it is important that this finding be reported as future research in this area may provide further illumination as to its cause.

### Study limitations

The findings in this study may be limited in their generalisability due to the fairly specific nature of the study sample group. Although recruitment was aimed at pregnant women from all SES backgrounds, we found that mainly women from a high SES population volunteered to take part. Despite their similar socio-economic status classification, there were a variety of women of different ethnic backgrounds, ages, nationalities and educational backgrounds who chose to take part in the study. The women were generally ‘comfortable’ in terms of household income and none were experiencing poverty or deprivation. There was a range of dietary patterns across the women taking part in the study from those that could be classed as relatively unhealthy (low fruit and vegetable intake; high levels of processed foods; low fibre, etc.) to those that could be classed as relatively healthy. The sample was unusual in reporting high breastfeeding rates and breastfeeding duration, compared with the general UK population. Further research should clarify whether the findings of this study can be generalised beyond the specific sample who took part in this study, although there is no reason to suggest it would not.

A further potential limitation of the study is that it accounted for DHA levels through use of a FFQ rather than a direct measure. However, this has the benefit of directly linking the findings of the study to maternal dietary intake rather than solely physiological measures. Using this method, any association that can be shown between dietary DHA intake (for example, with increased consumption of oily fish) and improved infant outcomes (for example, in relation to visual acuity) will be able to inform public health recommendations for pregnant women. As individual fatty acid metabolism will vary substantially, showing a direct link to changes in cognitive performance based on maternal dietary intake should allow dietary recommendations to be made which are more universal than those based on research linked solely to individual physiological fatty acid profiles (such as, for example, plasma-level measures). However, the ideal scenario, and one that could be employed in follow up studies, would involve concurrent use of a dietary intake measure, such as a FFQ, and physiological measurements such as blood plasma levels.

It is also important to take in account the postnatal diet when looking at factors which may affect infant cognitive development. While the focus of this study was on the prenatal diet we also collected data relating to postnatal diet and yet found no significant effects of postnatal DHA intake in relation to our outcome measures. More detailed examination of the postnatal diet would be ideal in future studies, for example looking at any differences between breastfed and formula fed infants on cognitive outcome measures. For this to occur we would likely need to collect more detailed post-natal dietary data and likely have less variation in the postnatal formula fed group, if they were being compared to a breast-fed group. This is certainly a continuation of the research reported here that would be worthwhile pursuing in the future.

## Conclusions

Putting together the findings from all of the tasks in this study, and considering them in relation to the inconsistent pattern of findings from previous research, it is impossible to conclude, with any reasonable degree of certainty, whether or not higher intake of DHA (either maternally during pregnancy or breastfeeding, or via infant postnatal levels) promotes better infant cognitive development. This view is supported by the most recent Cochrane reviews[43,44] reporting that, on balance, the current published empirical evidence does not support the theory of higher DHA levels resulting in better infant cognitive development (in infants born at term). Despite the number of positive studies reporting such a link, routine maternal supplementation and/or addition of DHA to infant formula should not be recommended at this time based on purported cognitive outcomes.

We also note that even in the case of visual acuity the effect we report is only apparent at 9 months post-partum, not at 4.5 months, when it may be expected that any differences between individuals may be more apparent. It is important to be aware that this may be a common feature in studies examining the impact of nutritional factors on development; namely, that the findings appear to be transient. However, this does not necessarily mean that they are any less important. Significant transient effects may well have consequences throughout the developmental course that are not initially apparent. Furthermore, transient findings support the rationale of considering examination of nutrient levels at more than one time point during pregnancy. Specific nutrients appear to have a critical point of action on the developing brain, likely related to the specific development within a particular cognitive system occurring at the time.

Finally, we feel it is with caution that findings of studies such as this are translated into advice for pregnant women regarding their diet and supplement choices. At the moment there appears to be no conclusive, unequivocal evidence to support the recommendation that pregnant women increase DHA levels for the purpose of optimising postnatal cognitive development in their infant. This is not to say that there may not be other, evidence based, reasons to do this (for example, in relation to allergy development; or for maternal cardiovascular health), however, in the case of cognitive or visual development10, the empirical support for higher prenatal intake is still lacking.

## Supporting information

S1 File PLoS one supporting information 1—Data file.sav. (SAV)
